# Supplementary material for: Adjusting for age improves identification of gut microbiome alterations in multiple diseases
Source: eLife. 2020 Mar 11;9:e50240. doi: 10.7554/eLife.50240 (PMC7065848; doi:10.7554/eLife.50240)
Supplement: Transparent reporting form [file elife-50240-transrepform.docx]

***eLife’s* transparent reporting form**

We encourage authors to provide detailed information *within their submission* to facilitate the interpretation and replication of experiments. Authors can upload supporting documentation to indicate the use of appropriate reporting guidelines for health-related research (see [EQUATOR Network](http://www.equator-network.org/%20)), life science research (see the [BioSharing Information Resource](https://biosharing.org/)), or the [ARRIVE guidelines](http://www.plosbiology.org/article/info:doi/10.1371/journal.pbio.1000412) for reporting work involving animal research. Where applicable, authors should refer to any relevant reporting standards documents in this form.

If you have any questions, please consult our Journal Policies and/or contact us: [editorial@elifesciences.org](mailto:editorial@elifesciences.org).

**Sample-size estimation**

- You should state whether an appropriate sample size was computed when the study was being designed
- You should state the statistical method of sample size computation and any required assumptions
- If no explicit power analysis was used, you should describe how you decided what sample (replicate) size (number) to use

Please outline where this information can be found within the submission (e.g., sections or figure legends), or explain why this information doesn’t apply to your submission:

The present study is a meta-analysis of previously published cohorts. The details of sample selection, group allocation and sample size estimation have been explained in the original studies which have been referred in the study. Therefore, the requirement for these information do not apply to the current submission.

**Replicates**

- You should report how often each experiment was performed
- You should include a definition of biological versus technical replication
- The data obtained should be provided and sufficient information should be provided to indicate the number of independent biological and/or technical replicates
- If you encountered any outliers, you should describe how these were handled
- Criteria for exclusion/inclusion of data should be clearly stated
- High-throughput sequence data should be uploaded before submission, with a private link for reviewers provided (these are available from both GEO and ArrayExpress)

Please outline where this information can be found within the submission (e.g., sections or figure legends), or explain why this information doesn’t apply to your submission:

As mentioned in the previous section, the present study is a meta-analysis of previously published cohorts. Supplementary Files 1 and 2 provided along with the Manuscript provide the details of the datasets used in the current study.

The key in-house source codes used in this meta-analysis have been provided as Supplementary File 4. The shotgun data of the ELDERMET is available for download from the ELDERMET website at <http://eldermet.ucc.ie/temp1/eldermet_shotgun_data_filtered_all_sample.tar>.

**Statistical reporting**

- Statistical analysis methods should be described and justified
- Raw data should be presented in figures whenever informative to do so (typically when N per group is less than 10)
- For each experiment, you should identify the statistical tests used, exact values of N, definitions of center, methods of multiple test correction, and dispersion and precision measures (e.g., mean, median, SD, SEM, confidence intervals; and, for the major substantive results, a measure of effect size (e.g., Pearson's r, Cohen's d)
- Report exact p-values wherever possible alongside the summary statistics and 95% confidence intervals. These should be reported for all key questions and not only when the p-value is less than 0.05.

Please outline where this information can be found within the submission (e.g., sections or figure legends), or explain why this information doesn’t apply to your submission:

The details of the statistical analyses have been described in the Methods section of the manuscript (along with brief summary in the Results section of the manuscript). Wherever possible, exact p-values have been reported, except in scenarios where the significance were used as a threshold for feature selection. In these as well as other cases, the p-value thresholds along with methods of multiple testing have been mentioned either in the Results, Methods section or in the Figure legends.

(For large datasets, or papers with a very large number of statistical tests, you may upload a single table file with tests, Ns, etc., with reference to sections in the manuscript.)

**Group allocation**

- Indicate how samples were allocated into experimental groups (in the case of clinical studies, please specify allocation to treatment method); if randomization was used, please also state if restricted randomization was applied
- Indicate if masking was used during group allocation, data collection and/or data analysis

Please outline where this information can be found within the submission (e.g., sections or figure legends), or explain why this information doesn’t apply to your submission:

The present study is a meta-analysis of previously published cohorts. The details of sample selection, group allocation and sample size estimation have been explained in the original studies. As such these details do not apply to the current submission.

**Additional data files (“source data”)**

- We encourage you to upload relevant additional data files, such as numerical data that are represented as a graph in a figure, or as a summary table
- Where provided, these should be in the most useful format, and they can be uploaded as “Source data” files linked to a main figure or table
- Include model definition files including the full list of parameters used
- Include code used for data analysis (e.g., R, MatLab)
- Avoid stating that data files are “available upon request”

Please indicate the figures or tables for which source data files have been provided:

The details of the datasets used in the current study have been uploaded as Supplementary Files 1 and 2. The source data used for Figure 2, Figure 4 and Figure 5 have been supplied as additional source data files for the corresponding figures. The protocol adopted in this study use publicly available R modules and softwares. The key in-house source codes used in this meta-analysis have been provided as Supplementary File 4. The shotgun data of the ELDERMET is available for download from the ELDERMET website at <http://eldermet.ucc.ie/temp1/eldermet_shotgun_data_filtered_all_sample.tar>.
